# On comparison of net survival curves

**DOI:** 10.1186/s12874-017-0351-3

**Published:** 2017-05-02

**Authors:** Klemen Pavlič, Maja Pohar Perme

**Affiliations:** University of Ljubljana, Faculty of Medicine, Institute for Biostatistics and Medical Informatics, Vrazov trg 2, Ljubljana, 1000 Slovenia

**Keywords:** Relative survival, Net survival, Log-rank test, Regression model

## Abstract

**Background:**

Relative survival analysis is a subfield of survival analysis where competing risks data are observed, but the causes of death are unknown. A first step in the analysis of such data is usually the estimation of a net survival curve, possibly followed by regression modelling. Recently, a log-rank type test for comparison of net survival curves has been introduced and the goal of this paper is to explore its properties and put this methodological advance into the context of the field.

**Methods:**

We build on the association between the log-rank test and the univariate or stratified Cox model and show the analogy in the relative survival setting. We study the properties of the methods using both the theoretical arguments as well as simulations. We provide an R function to enable practical usage of the log-rank type test.

**Results:**

Both the log-rank type test and its model alternatives perform satisfactory under the null, even if the correlation between their p-values is rather low, implying that both approaches cannot be used simultaneously. The stratified version has a higher power in case of non-homogeneous hazards, but also carries a different interpretation.

**Conclusions:**

The log-rank type test and its stratified version can be interpreted in the same way as the results of an analogous semi-parametric additive regression model despite the fact that no direct theoretical link can be established between the test statistics.

**Electronic supplementary material:**

The online version of this article (doi:10.1186/s12874-017-0351-3) contains supplementary material, which is available to authorized users.

## Background

Relative survival analysis is a subfield of survival analysis with competing risks, where cause-specific information is of interest, but the types of events are unknown. The most common example is the cancer registry data, which contains information on survival for patients diagnosed with cancer, but has no reliable cause of death information available. Since the proportion of non-cancer related deaths is considerable, cancer-specific analysis is of interest.

To overcome this issue, additional assumptions are introduced in the field: we assume that the causes of death can be split into ‘disease’ and ‘other’ and we assume that the hazard of dying of ‘other’ causes of the observed patients is comparable to the mortality of their counterparts in the general population that do not have the disease. Further, we assume that the effect of mortality due to the disease in question on the total mortality of the country’s population is negligible. Under these assumptions, we can use the population mortality tables to determine the ‘other’ cause mortality of our patients and hence deduce the excess mortality due to the disease in question.

Several measures may be of interest in the relative survival field. When interested in the excess (disease specific) hazard, one may wish to first estimate the survival curve associated with it and then to compare it between subgroups and possibly use a regression model to study it further. The focus of our paper is the recently introduced log-rank type test [1] that compares net survival between subgroups.

Our motivating example comes from a study of survival of patients after acute myocardial infarction (AMI) which was carried out at the University Clinical centre in Ljubljana, Slovenia, see [2] for example. Being interested in the excess mortality that these patients experience due to the infarction, we focus on a subgroup of 494 patients, aged between 45 and 75, who were recruited from 1984 to 1986 and followed for 10 years. The patients were included in the study at the time of release from the hospital after an infarction, the end point was death of any cause, whereas cause-of-death information was not available. Several variables were recorded at the time of admission, but we focus here on sex and age only. In this work, we are not interested in the overall mortality of these patients, but rather in the excess hazard that can be attributed to their cardio-vascular disease and the effect of sex on it. The net survival curves with respect to sex are markedly different and the newly introduced log-rank type test [1] reports a significant difference (*p*=0.02). However, the authors of this method warn that this test assumes homogeneous hazards for all individuals within each group, an assumption which is obviously violated in our case, where patients of each gender differ substantially with respect to age which most likely affects also their excess hazard. The stratified test using age in 5-year intervals indicates that the difference between males and females is no longer statistically significant (*p*=0.09). One could resort to a parametric regression model with sex and age as covariates instead, but for that purpose, further assumptions about the baseline hazard are needed. Two semi-parametric approaches to modelling (that do not require any additional assumptions about the baseline hazard) have also been proposed [3, 4], but have not been used much in practice and their properties have not been examined in depth. Furthermore, the possibility to stratify with respect to a covariate has not yet been implemented with these models, whilst introducing age as an additional covariate requires additional assumptions. It is thus not clear to what extent these methods are comparable and what is the method of choice to answer our question.

In this paper we investigate the properties of the non-stratified and stratified version of the test (as defined in [1]) and give advice for its usage. The goal of the paper is to simplify the understanding of the new methodology by paralleling it with the related existing methods both in classical survival framework and within the net survival setting: we build on the association between the log-rank test and the univariate or stratified Cox model and explain how the story changes when comparing net survival curves. With this approach we wish to provide a clear place for the new method among the existing ones, by providing an efficient implementation in R, we wish to make it directly usable.

## Methods

Let $\widetilde {T}_{E,i}$, $\widetilde {T}_{P,i}$ and *C*_*i*_ denote the time to death due to the disease in question, time to death due to other reasons and time to censoring of *i*th individual, respectively. We assume that the censoring times *C*_*i*_ are independent of $\widetilde {T}_{P,i}$ and $\widetilde {T}_{E,i}$. Only one of these times may be observed for each individual, we denote it by ${T_{i}}=\min (\widetilde {T}_{P,i},\widetilde {T}_{E,i},C_{i})$. Assuming this framework, our data, in which the cause of death is unknown, consists of pairs (*T*_*i*_,*δ*_*i*_), where *δ*_*i*_ is the censoring indicator that equals 1 in case of death and 0 in case of censoring.

Denote the hazard of *i*th individual as *λ*_*O*,*i*_(*t*), the subscript *O* indicating that this is the overall hazard whose effect we can observe. We assume that this hazard can be split into two additive components - the hazard due to the disease *λ*_*E*,*i*_ (excess hazard due to the disease) and the hazard due to other causes *λ*_*P*,*i*_:

1 $$\begin{array}{@{}rcl@{}} \lambda_{O,i}(t)=\lambda_{E,i}(t) + \lambda_{P,i}(t)  \end{array} $$

We further assume that for each individual *i*, the value of hazard of other causes can be read from the national population mortality tables for age, sex and calendar year of diagnosis of the *i*th individual. It is hence often referred to as the population hazard and denoted by subscript *P*.

In this work, our interest lies in *λ*_*E*_ and we are interested in net survival, i.e. the survival curve that depends solely on *λ*_*E*_, $S_{E}(t)=\exp \left \{-\int _{0}^{t}\lambda _{E}(u)du\right \}$. The ideal data to estimate this quantity would be the pairs (*T*_*E*,*i*_,*δ*_*E*,*i*_), where *T*_*E*,*i*_ is the possibly censored time to death from the disease for each individual (${T_{E,i}}=\min (\widetilde {T}_{E,i},C_{i})$) and *δ*_*E*,*i*_=1 if $\widetilde {T}_{E,i}\leq C_{i}$ and 0 otherwise. These data cannot be available in real world, where there is no way to exclude other causes, but we can use it in theory and in simulations to better understand the properties of the log-rank type test. We shall refer to it as the *hypothetical world* data, as opposed to the *real world data* (*T*_*i*_,*δ*_*i*_). The real world data in our case present the competing risks setting from which we wish to extract information about *λ*_*E*_, whereas the hypothetical world data present the simpler framework where patients are subject only to one hazard and thus the basic survival analysis methods (Kaplan-Meier, Cox model) can be directly used.

With the hypothetical world data, the classical log-rank test can be used to compare the net survival curves with respect to a covariate *X* which splits the data into *k* groups. The null hypothesis states that *S*_*E*,1_(*t*)=…=*S*_*E*,*k*_(*t*), or alternatively *λ*_*E*,1_(*t*)=…=*λ*_*E*,*k*_(*t*).

***Hypothetical world data***

Using the counting process notation, we let *N*_*E*,*i*_(*t*) denote the counting process for individual *i*: $N_{E,i}(t)=I(T_{E,i}\leq t, \widetilde {T}_{E,i}\leq C_{i})$ and *Y*_*E*,*i*_(*t*) denote the at risk process for each individual: *Y*_*E*,*i*_(*t*)=*I*(*T*_*E*,*i*_≥*t*). Further, we use *N*_*E*,*h*_(*t*) and *Y*_*E*,*h*_(*t*) for the sum of *N*_*E*,*i*_ and *Y*_*E*,*i*_ for all individuals *i* belonging to each of the subgroups *h*=1,…,*k*. The test statistic compares the observed and the expected number of events in group *h* at each time point. The observed number of events at each time *u* is denoted as *d* *N*_*E*,*h*_(*u*), the expected number of events is calculated from the total number of deaths at that time(*d* *N*_*E*,·_(*u*)) as the proportion corresponding to the ratio between the number of individuals at risk in group *h* (*Y*_*E*,*h*_(*u*)) and the total number of individuals still at risk (*Y*_*E*,·_(*u*)):

2 $$\begin{array}{@{}rcl@{}} Z_{h}(\tau) \,=\,\! \int_{0}^{\tau}\! I\!\left(Y_{E,\cdot}(u)\! >\!0\right)\! \left[\!dN_{E,h}(u) \,-\, \frac{Y_{E,h}(u)}{Y_{E,\cdot}(u)} dN_{E,\cdot}(u)\!\right]\!,  \end{array} $$

where *τ* is the follow-up time. The test statistic is then calculated as

$$U=Z^{T}\widehat{\Sigma}^{-1}Z, $$

where *Z*=(*Z*_1_(*t*),…,*Z*_*k*−1_(*t*))^′^ and $\widehat {\Sigma }$ is the estimated variance-covariance matrix, see [5] for details. Under the null hypothesis, the test statistic can be shown to be asymptotically $\chi ^{2}_{k-1}$ distributed.

The derivation of the log-rank test requires the hazard *λ*_*E*,*h*,*i*_ to be equal for all individuals *i* in a certain group *h*, we shall refer to this property as the homogeneity of the hazards. If this is not true and there is another categorical variable *S* which explains the differences within each subgroup, one can use the stratified log-rank test, where the homogeneity property is required to hold only within the strata of each group. We calculate the *Z*_*h*_ value in each stratum separately

3 $$\begin{array}{@{}rcl@{}} Z_{h,s}(\tau) &=& \int_{0}^{\tau} I\left(Y_{E,\cdot,s} (u) > 0 \right)\\ &&\times\left[ dN_{E,h,s} (u) - \frac{Y_{E,h,s}(u)}{Y_{E,\cdot,s}(u)} dN_{E,\cdot,s}(u) \right]  \end{array} $$

and then sum over all *m* strata to get the test statistic (see [5] for more details)

$$U=\left(\sum_{s=1}^{m}Z_{s}\right)^{T} \left(\sum_{s=1}^{m}\widehat{\Sigma}_{s}\right)^{-1}\left(\sum_{s=1}^{m}Z_{s}\right), $$

where *Z*_*s*_=(*Z*_1,*s*_(*t*),…,*Z*_*k*−1,*s*_(*t*)).

***Real world data*** With the real world data, we wish to test the same null hypothesis of equal excess hazards, but the number of cause-specific deaths (*d* *N*_*E*,*h*_ in formula Eq. (2)) and the number at risk in the hypothetical world (*Y*_*E*,*h*_ in formula Eq. (2)) cannot be directly observed. We thus have to estimate it by the help of population tables, we shall denote these estimates by $\widehat {dN}_{E,h}$ and $\widehat {Y}_{E,h}$ respectively. Let (*N*_*i*_,*Y*_*i*_) denote the counting process defined as above from the observed data (*T*_*i*_,*δ*_*i*_). We use these data and merge it with the population mortality data to estimate the number of deaths due to the disease in question (see [6] for details):

4 $$\begin{array}{@{}rcl@{}} \widehat{dN}_{E,i}(t)=\frac{dN_{i}(t)}{S_{P,i}(t)} - \int_{0}^{t} \widehat{Y}_{E,i}(u)\lambda_{P,i}(u)du,  \end{array} $$

where $\widehat {Y}_{E,i}(t)=\frac {Y_{i}(t)}{S_{P,i}(t)}$, and *S*_*P*,*i*_(*t*) and *λ*_*P*,*i*_(*t*) are the population survival and hazard for *i*th individual which are obtained from the population mortality tables. In this estimation, we follow the idea of the PP estimator (Pohar Perme estimator) [6]: the total number of events must be diminished by the number of expected deaths in the population (second term on the right of (4)) and both the observed number of deaths and number at risk must be weighted (by *S*_*P*,*i*_) to properly represent the numbers we would observe in the hypothetical world where no one dies of *λ*_*P*,*i*_. Therefore, the analogous test statistic is calculated using [1]:

5 $$\begin{array}{@{}rcl@{}} Z_{h}(\tau) &=& \int_{0}^{\tau} I \left(\widehat{Y}_{E,\cdot} (u) > 0 \right)\\ &&\times\left[ \widehat{dN}_{E,h} (u) - \frac{\widehat{Y}_{E,h}(u)}{\widehat{Y}_{E,\cdot}(u)} \widehat{dN}_{E,\cdot} (u)\right] \end{array} $$

Similarly, the stratified version is calculated as

6 $$\begin{array}{@{}rcl@{}} Z_{h,s}(\tau) &=& \int_{0}^{\tau} I \left(\widehat{Y}_{E,\cdot,s} (u) > 0 \right)\\ &&\times\left[ \widehat{dN}_{E,h,s} (u) - \frac{\widehat{Y}_{E,h,s}(u)}{\widehat{Y}_{E,\cdot,s}(u)} \widehat{dN}_{E,\cdot,s} (u)\right] \end{array} $$

### Properties and interpretation of the log-rank type test

The paper by Graffeo et al. [1] defined the test statistic and derived its distribution under the null hypothesis. They used simulations to illustrate the asymptotically proven properties in practice and showed proper behaviour under the null hypothesis as well as reasonable power under different alternatives. The log-rank test on hypothetical world data and the log-rank type test on real world data have the same null hypothesis and differ only due to the different data available. We can therefore expect similar behaviour, but a smaller power in the case of real world data, where some of the information cannot be observed. Since the interest lies in the comparison of net survival, which is defined by the excess hazard part of the model (1), we can expect the proportion of events due to excess hazard to crucially affect the power.

#### Log-rank test and regression models

To further understand the properties and the interpretation of the log-rank type test we compare it to its main alternative - a regression model. The standard log-rank test statistics comparing two groups defined by the binary covariate *X* is identical to the score test statistic in a univariate Cox model containing covariate *X* (when no ties are present) [7]. Therefore, the two approaches have the same properties and can be interpreted in the same way. This means that with hypothetical world data, the score test statistic of the null hypothesis *H*_0_:*β*_*X*_=0 in the Cox model

7 $$\begin{array}{@{}rcl@{}} \lambda_{E}(t,x)=\lambda_{E_{0}}(t)e^{\beta_{X} x}  \end{array} $$

is identical to the log-rank test statistic. The analogous model with the real world data (*T*_*i*_,*δ*_*i*_) is the additive model

8 $$\begin{array}{@{}rcl@{}} \lambda_{O}(t,x)=\lambda_{P}(t,x) + \lambda_{E_{0}}(t)e^{\beta_{X} x}  \end{array} $$

Again, the null hypothesis *H*_0_:*β*_*X*_=0 is of interest and this null hypothesis is clearly equivalent to the null hypothesis of the log-rank type test, i.e. *λ*_*E*,1_(*t*)=*λ*_*E*,2_(*t*), where the two groups are defined by the binary covariate *X*.In order to expect similar behaviour of the additive model and the log-rank type test when testing this hypothesis, the assumptions required by both methods should also be the same. This implies that the $\lambda _{E_{0}}(t)$ in Eq. (8) should be left completely unspecified, since the log-rank type test also makes no distributional assumption within each group. Unfortunately, the idea of using partial likelihood in the framework of the relative survival does not work, since the baseline hazard does not cancel out (due to *λ*_*P*_ in Eq. (8)). Therefore, the $\lambda _{E_{0}}(t)$ in model Eq. (8) is usually defined as a function of a few parameters - it has been initially defined with a stepwise constant hazard function [8] and many alternative parametric specifications have been proposed since [9–11]. The fully parametric model specified in this way can be fitted using maximum likelihood. On the other hand, only two semi-parametric approaches allowing $\lambda _{E_{0}}(t)$ to be left unspecified have been proposed [3, 4]. Both approaches require some smoothing when fitting (hence making some weak assumptions about the baseline hazard form), therefore, these models cannot be expected to give identical results as the log-rank type test either. It can be quickly seen that the test statistics and the *p*-values are not identical, therefore, the question is to what extent the log-rank type test and the regression models behave in the same way and can be interpreted in the same way.

#### The stratified vs the non-stratified version

The derivation of the distribution of the log-rank test statistic under the null hypothesis requires *λ*_*E*_(*t*) to be equal for individuals within a certain subgroup defined by *X* [1]. This may not be true in practice, hence a stratified version is proposed to take this inhomogeneity into account. The stratified log-rank test implies that the groups formed by *X* are not compared on the whole sample but rather in strata defined by a covariate *S*. Then, the results from all strata are pulled together to form a single test statistic value. The direct analog of this approach with the hypothetical world data is the stratified Cox model

$$\begin{array}{@{}rcl@{}} \lambda_{E}(t,x,s)=\lambda_{{E_{0}},s }(t)e^{\beta_{X} x} \end{array} $$

in which the baseline hazard is allowed to differ between strata, but a common coefficient *β*_*X*_ describing the effect of *X* is estimated. In the hypothetical world, where the framework of the classical survival analysis is used, the stratified log-rank test and the score test of the stratified regression model give identical results. On the other hand, the estimation of *β*_*X*_ in a Cox model stratified by *S* can be compared to a multivariate model containing both *X* and *S*. If the covariate *S* satisfies the proportional hazards assumption (i.e. $\lambda _{E_{0},s}(t)=\lambda _{E_{0}}(t)\exp (\beta _{S} s)$), *β*_*X*_ has the same interpretation with both the stratified and the multivariate model. Note that if *X* and *S* are not independent, the equality between the stratified and the multivariate regression model implies that the interpretation of the stratified model is importantly different from the non-stratified version (i.e. univariate model).

To draw the analogy further, say we wish to compare two groups defined by *X* with the hypothetical world data. If hazards within these groups are not homogeneous, but depend on *S*, the data follow a model with both covariates (*X* and *S*) and the Cox model fit will not be perfect if *S* is omitted. The estimated coefficient for *X* will shrink toward zero, and the power for testing the null hypothesis *H*_0_:*β*_*X*_=0 shall be lower. Since the Cox model score test statistic and log-rank test statistic are equal, loss of power shall also occur with the log-rank test if *S* is not taken into account and the non-stratified version of the test is used. The same can be then expected also when using the relative survival methodology.The stratified log-rank type test may therefore be used for two reasons: to correct for the fact that hazard is not homogeneous within subgroups and to compare subgroups conditional on a second covariate *S*.

### Simulations

Based on theoretical relationships given in the previous sections, we can formulate two main issues to be explored with simulations:

- How does the log-rank type test relate to the additive model? We know that the two tests address the same null hypothesis, but their test statistics are not the same. The questions to be answered are: can we expect the same size under the null hypothesis, do the tests have the same power with different alternatives?
- Can the non-stratified version of the log-rank type test be used even if comparing groups with non-homogeneous hazards? How does the homogeneity assumption affect the size of the test, how is the power affected, when should the stratified test be used instead?

#### Simulation design

The scenarios of all simulations have some common properties, that will allow for clear comparisons:

- We fix the data set size to 1000, since small sample behaviour is not an issue of interest in this paper. 5000 simulation runs are performed in each scenario.
- We simulate the times following the competing risks model (8) by first simulating the latent times ($\widetilde {T}_{E}$, $\widetilde {T}_{P}$ and *C*) and then taking the respective minima. This enables us to generate both the real world as well as the hypothetical world data.
- Several covariates enter the model. The population part always depends on demographic variables, we use age, sex and calendar year and denote them by *D*. The excess hazard part can depend on *D*, but also on other variables *Z*, the vector of all covariates will be denoted by (*D*,*Z*). Some of these variables will be used as *X* and *S* in our simulations. All variables are generated independently of each other.
- We use an exponential model to simulate the times $\widetilde {T}_{E}$, $\lambda _{E}(t,x)=\lambda e^{\beta _{X} x}\phantom {\dot {i}\!}$, i.e. the baseline excess hazard is fixed in time.
- We use the Slovene population tables and match them with the demographic variables to simulate the times $\widetilde {T}_{P}$.
- We censor all the individuals after 10 years of observation. We do not censor any data before that time since censoring is not a crucial issue we would like to research.
- The real world data is formed by $T_{i}=\min (\widetilde {T}_{E,i},\widetilde {T}_{P,i},C_{i})$, $\delta _{i}=I(\widetilde {T}_{E,i}<C_{i} \vee \widetilde {T}_{P,i} < C_{i})$
- The hypothetical world data is formed by $T_{E,i}=\min (\widetilde {T}_{E,i},C_{i})$, $\delta _{E,i}=I(\widetilde {T}_{E,i}<C_{i})$.

**Fig. 1:**
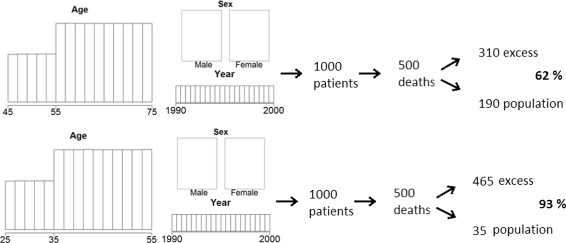
Simulation design
- To make the two scenarios comparable in real world data, we set the baseline excess hazard $\lambda _{E_{0}}$ to get a similar overall number of deaths - approximately half of the individuals die of any cause in the period of observation (10 years).

Since both the log-rank type test and the additive regression model address the same question defined in the hypothetical world, any difference that may be observed between the two methods must come from the different amount of information available in the real world. We thus design the simulations in a way that allows us to carefully study these differences. Note that the simulations are not attempting to perfectly reflect the data we might meet in practice (we believe this is an endless task that provides very little understanding), instead they try to address the various situations which may cause differences in the test statistics. All the simulations are simplified so that the cause for the properties can be tracked and different grades of effects are used in several cases to show how a certain property gains importance. Figure 1 presents the parameters for the two simulation scenarios we consider:

- We use two distributions for demographic variables *D*: sex is always balanced, calendar year is uniform between 1990 and 2000, age can be either between 45 and 75 or between 25 and 55, in both cases we use a mixture of two uniform distributions to get more older patients, see Fig. 1. The first distribution is a simplified version of our motivating example. The demographic variables determine population mortality hazard *λ*_*P*_.

The main factor we have thus changed in the two scenarios is the importance of the excess hazard *λ*_*E*_ compared to the population hazard *λ*_*P*_. We measure this importance with the proportion of deaths due to excess hazard, i.e. the proportion of patients that died (*δ*_*i*_=1) in which $\widetilde {T}_{Ei}<\widetilde {T}_{Pi}$, with our parameters the two proportions are approximately 62% and 93%. The first scenario presents a simplified version of what we could expect in practice, the second is used to show how the results converge to those of the hypothetical world when the excess hazard becomes high compared to population hazard. A third scenario with 41% of patients dying due to excess hazard (age between 50 and 80, mixture of two uniform distributions as above) is added to show that the results follow the same logic even with lower proportions, the detailed results for this scenario are reported in the Additional file 1.

In terms of the covariates *X* and *S*, the main differences in the real world when compared to the hypothetical world could arise from the fact that a covariate is or is not in the population tables (we denote this by *X*∈*D* or *X*∉*D*, respectively). We also change the number of groups defined by both covariates and, in case of binary variables, make their distribution balanced (the groups occurring with equal probability, denoted as *bal*) or imbalanced (the group with the higher value of X occuring with probability 0.9).

We simulate data so that both the real world and the hypothetical world data are available and compare four different test statistics.

- The log-rank type test (denoted as *LRt*) calculated on the real world data.
- The results of a coefficient test in the semi-parametric additive model (*sAM*). The EM model [3] was chosen since the functions are readily available in the relsurv [12] package. The Wald test is used in all cases.
- The results of a Wald test for a coefficient in a fully-parametric additive model (*fAM*) by (8). This model is modelled as in [8] with $\lambda _{E_{0}}$ being constant. It is used as the reliable option since the semi-parametric models have not been used much in practice. We use it primarily to double-check our simulations. Its results are not directly comparable to *LRt* in terms of power since it works under the additional assumption that the baseline excess hazard is constant (which is true in our simulations).
- The log-rank test (denoted as *LRh*) calculated on the hypothetical world data. This test is used as an ideal situation to which the other tests can only approach. It is also used to double-check our simulations. The results of the Cox model coefficient test in the hypothetical world are not reported, since they are practically the same.

Technical note: Wald test is used for practical reasons - it is available in the software for both semi-parametric and fully parametric additive model. It is also asymptotically equivalent to the score test and the differences should be minimal since we are using large samples.

### The function rs.diff and its usage

To ensure straightforward usability of the log-rank type test, we provide a function named rs.diff which we include in the package for relative survival analysis relsurv [12] that is readily available from CRAN [13]. This function directly mimics the function for the calculation of the classical log-rank test survdiff, which is available in the survival package [14]. The main difference between the syntax of the two functions is in the usage of the population tables, where we follow the syntax of the other functions of the relsurv package that rely on the object ratetable (provided in the survival package). For example, take a data set data1, in which the first three lines equal:



where time denotes the time *T*_*i*_ in days, cens denotes the censoring status *δ*_*i*_, age is the age of the patients in years, sex denotes the gender (1= male, 2=female) and diag denotes the date of diagnosis in date format which counts the number of days since January 1st, 1960. The log-rank test comparing the overall survival curves with respect to sex is run as



and the log-rank type test comparing the net survival curves with respect to sex is run as



Here, the ratetable object in the formula part ensures that the demographic variables are in the right format (age has to be in days, the calendar year is denoted as year in the population tables and as diag in the data) and the ratetable argument tells that the Slovene population tables should be used for the analysis. A test stratified by covariate *X* can in both cases be called by adding an object named strata(X) into the formula.

While the syntax is intentionally similar with the two tests, there is an important difference in the calculation of the two test statistics. The integral in Eqs. (2) or (3) can be written as a simple sum at each event time since both *Y* and *N* are step-wise constant functions.

However, the calculation of the log-rank type test comparing the net survival curves is not that straightforward. The reason lies in the fact that the integral in Eqs. (5) and (6) cannot be written as a simple sum at event times, the reason for this stems from the integral in the term Eq. (4). This integral is a continuous function in time and is non-zero also at times when there is no event. Therefore, the integral of $\widehat {dN}_{E}$ cannot be written as a finite sum. Furthermore, the *λ*_*Pi*_ values are piecewise constant, but they change at different times *t* for different individuals (they change on the first of January each year and when the patient gets a year older), so this cannot be easily solved. Therefore, a simplification is used in our function - we calculate the integral in daily intervals, in which *λ*_*Pi*_ is constant and the replacement of the integral by a sum causes negligible effect. Nevertheless, we cannot avoid the additional computational intensity added by the term Eq. (4), since the *λ*_*Pi*_ values must be read from the population tables for all individuals at all times while still at risk. We use a C++ routine to make the R functions faster, but the rs.diff function remains slower than the survdiff function. It should be noted however, that the same problem arises with the semi-parametric additive regression model (and the net survival curve estimation): while the fully parametric models only require the knowledge of *λ*_*P*_ at the time of death of each individual, the values at all times at risk must be obtained for the semi-parametric model. The intuition behind this important difference is yet to be fully understood. A similar problem of continuity exists also for the traditional estimators of net survival (Ederer II), but has been entirely overlooked in the literature.

## Results

### Simulation results

In this section we present the simulations results. A large scale simulation study has been performed but only the results that bring interesting insight are reported in the tables.

#### Log-rank type test and regression - comparison of the size

First, we compare the behaviour of the log-rank type test with that of the additive model. We start with a comparison of the size of the two tests under the null hypothesis. In Table 1 the results of several tests are compared. For each test, we report the proportion of simulation runs in which the null hypothesis was rejected, i.e. the *p*-value was below 0.05.

**Table 1:** Comparison of the log-rank type test and the additive model: size

	62% events due to ex. haz.				93% events due to ex. haz.			
	*LRt*	*sAM*	*fAM*	*LRh*	*LRt*	*sAM*	*fAM*	*LRh*
*X*∈*D*, bal, bin (sex)	0.046	0.050	0.045	0.052	0.047	0.049	0.049	0.047
*X*∉*D*, bal, bin	0.049	0.052	0.046	0.053	0.047	0.048	0.048	0.049
*X*∉*D*, imbal, bin	0.050	0.053	0.050	0.052	0.049	0.045	0.045	0.047
*X*∉*D*, bal, 4 grps	0.052	0.052	0.041	0.050	0.046	0.046	0.046	0.047

Methods included: log-rank type (*LRt*), semi-parametric additive model (*sAM*), fully parametric additive model (*fAM*), log-rank test in hypothetical world (*LRh*). ((im)bal = (im)balanced variable, i.e., the groups occur with (un)equal probabilities; bin= binary variable; 4 grps = a variable with four groups). *X* is the categorical covariate of interest, *D* denotes the demographic variables

**Results** We can see (Table 1) that the size of the log-rank type test (*LRt*) is close to nominal and does not seem to be liberal in in any of the scenarios. The same can be claimed for the fully parametric model (*fAM*) and the semi-parametric model (*sAM*), the only exception is the scenario with only 41% of deaths due to excess hazard, where the size of the latter can be above its nominal value (a problem noted already in [3]). The log-rank test in the hypothetical world (*LRh*) serves as a check that the simulations are properly conducted.

**Correlation of the** ***p*** **-values:** While the sizes of both the log-rank type test and of the semi-parametric additive model are acceptable, the actual *p* values do not coincide as well. The correlation of the *p* values of the *LRt* and *sAM* in the above examples in the left part of the table is around 0.72. When the proportion of excess hazard deaths becomes large (right part of the table), both tests’ results become more similar to their versions in the hypothetical world (which are equal), hence the correlation in all the above examples in the right part of the table is above 0.98. The low correlation implies that one has to make a choice between which test to perform (both *LRt* and *sAM* reject the null hypothesis in around 3% of the cases, at least one of the two tests rejects the null hypothesis in 7% of the cases).

#### Log-rank type test and regression - comparison of the power

**Comments on the simulation scenarios:** Since we know that both tests simplify to the same test statistic in the hypothetical world, we can expect them to respond to the same alternative hypotheses also with the real world data. This reasoning is checked with simulations reported in Table 2. We look at several situations, in particular, we add two situations (last two rows in the table) where the effect of *X* on excess hazard is not constant in time and hence the proportional excess hazards assumption of the additive model is not met. We simulate crossing hazards, first in a situation where the overall effect is approximately 0 (*β* starts at 0.5 and changes to −0.5), and second, in a situation where the overall effect is similar as in other simulations (*β* starts at 0 and changes to 1).

**Table 2:** Comparison of the log-rank type test and the additive model: power

	62% events due to ex. haz.				93% events due to ex. haz.			
	*LRt*	*sAM*	*fAM*	*LRh*	*LRt*	*sAM*	*fAM*	*LRh*
*X*∈*D*, bal, bin (sex)	0.499	0.561	0.552	0.786	0.878	0.874	0.877	0.903
*X*∉*D*, bal, bin	0.487	0.578	0.567	0.792	0.877	0.877	0.876	0.903
*X*∉*D*, imbal, bin, ef >0	0.467	0.391	0.359	0.700	0.857	0.793	0.792	0.847
*X*∉*D*, imbal, bin, ef <0	0.537	0.720	0.711	0.864	0.888	0.931	0.933	0.943
*X*∉*D*, bal, 4 grps	0.339	0.408	0.385	0.629	0.736	0.743	0.745	0.787
*X*∉*D*, bal, bin, NPH, ef ≈0	0.052	0.074	0.062	0.047	0.048	0.053	0.051	0.049
*X*∉*D*, bal, bin, NPH	0.524	0.510	0.476	0.819	0.880	0.865	0.861	0.912

Methods included: log-rank type (*LRt*), semi-parametric additive model (*sAM*), fully parametric additive model (*fAM*), log-rank test in hypothetical world (*LRh*). ((im)bal = (im)balanced variable, i.e., the groups occur with (un)equal probabilities; bin= binary variable; 4 grps = a variable with four groups; ef = variable’s effect; NPH = nonproportional effect). *X* is the categorical covariate of interest, *D* denotes the demographic variables

In all cases, the power of the regression models is expected to be higher than the power of log-rank type test - regression models work with additional assumptions that are true in our scenarios. We are hence more interested in whether the difference between the log-rank type tests and the regression models is similar across different scenarios, if it is not, we could say that the tests are not susceptible to the same alternative hypotheses and hence have a different interpretation.

**Results** Several results can be read from Table 2:

- As expected, the semi-parametric model test in the 62% case has more power than the log-rank type test in most scenarios. This may be at least partly attributed to the additional implicit assumption of the semi-parametric model ($\lambda _{E_{0}}$ is smooth), which is true in our simulation scenarios ($\lambda _{E_{0}}$ is taken as a constant in simulations). Interestingly, the power of the fully parametric model is not higher than the power of the semi-parametric model, though it works with the additional information that the baseline hazard is constant throughout the interval.
- With the 62% case, the difference between the power of the log-rank type test and the semi-parametric model is similar in all cases of proportional hazards and balanced covariates. As the proportion of excess hazard deaths increases, the power of all tests becomes similar.
- Holding other simulation parameters equal, the power changes in the case of imbalanced groups. When the more common group has a lower hazard (*e* *f*<0), the power of any test gets higher, the opposite effect on power can be seen when patients in the more common group have a higher hazard. Interestingly, this effect seems more pronounced with the regression models than with the log-rank type test, an explanation for this may be the results of the 41% scenario: with an extremely low number of events due to excess hazard, the model fitting procedure does not converge, leading to huge variances and unreliable results (see Additional file 1: Table S4). The log-rank test thus seems the more stable and reliable option.
- When the proportionality assumption fails, the power of log-rank type test and the regression models becomes very similar, indicating that the tests not only have the same null hypotheses but also follow the same logic which makes them susceptible to the same alternatives. For example, none of methods can detect crossing hazards when the average effect is 0.

Based on these results, we can conclude there is no important difference between the interpretation of the log-rank type test and the test of a coefficient in a univariate additive model, but the power in the individual scenarios may be higher with the models if their additional assumptions are met.

#### Further notes on the power of log-rank type test

**Comments on the simulation scenarios:**

The excess hazard mortality is a crucial factor for the power of the tests in the hypothetical world, however, the power in the real world depends also on the proportion of the hypothetical world deaths that we actually observe. The population hazard can thus bee seen as a nuisance factor. To illustrate this fact with simulations we consider two scenarios (columns A and B in Table 3) with equal distribution of *D* (and hence equal *λ*_*P*_ values) and equal baseline excess hazard ($\lambda _{E_{0}}$) values. Working with a centered covariate (age) and changing only the sign of its effect (|*β*_*AGE*_| remains equal in both scenarios), we get two scenarios with equal power in the hypothetical world. In column A, the effect of age is in the same direction as in the population mortality tables (older individuals have a higher population and excess mortality hazard), in column B, the effect works in the opposite direction. We calculate the proportion of individuals who die due to excess hazard (*T*_*Ei*_<*T*_*Pi*_) among all individuals who die in the hypothetical world (*T*_*Ei*_<*τ*) (‘Observed proportion of excess hazard deaths‘ in Table 3). We then add a third scenario, where this proportion is held equal, but the age of individuals is lowered and hence the total number of population deaths is lower.

**Table 3:** Results of the log-rank type test (*LRt*) when varying the proportion of excess hazard deaths observed in real world data among all excess hazard deaths in hypothetical world data (’observed proportion’)

	A	B	C
Proportion with *T*_*P*_<10	0.34	0.34	0.29
Proportion with *T*_*E*_<10 (1- net survival)	0.35	0.35	0.35
Effect of age	*β*>0	*β*<0	*β*>0
Proportion of patients that die in 10 years	0.55	0.58	0.52
Proportion of excess hazard deaths among all deaths	0.51	0.51	0.57
Observed proportion of all excess hazard deaths	0.83	0.86	0.86
Power of *LRh*	0.934	0.933	0.935
Power of *LRt*	0.507	0.538	0.572

For these simulations, age was considered as the covariate in question as it has the largest effect on population mortality hazard and changing the direction of its effect can thus make an observable difference. We simplified its distribution and considered a binary covariate (55 or 75 years with 50% probability in columns A and B and 53 and 73 years in column C). Therefore, the proportion of excess deaths among all deaths is no longer equal to 62%. Note that fixing the mortality in the hypothetical world and in the population, scenarios that provide larger differences could not be designed. We thus repeated the simulations 50000 times, to guarantee that the differences are not a consequence of random variation.

**Results**

Table 3 confirms the importance of the amount of information lost in the real world, compared to the information available in the hypothetical world. This cannot be directly estimated with the real world data, but the direction of the covariate effect can serve as a guideline. The power in column B of Table 3 is higher as the power in column A, since the proportion of individuals who die due to excess hazard in the real world data is higher (86% of all hypothetical world deaths compared to 83% in column A). In fact, the columns A and B also differ in the total number of deaths (more deaths in column B), column C is added to prove that the observed difference in power is not due to the total number of deaths - with equal observed proportion of excess hazard deaths, a lower number of individuals dying due to other causes increases the power.

#### Non-stratified and stratified log-rank type test - comparison of the size

We now turn to the comparison of the stratified and non-stratified version. While we need the homogeneity assumption in theory, we would like to evaluate how important this assumption is in practice.

**Comments on the simulation scenarios:** We simulate scenarios with two covariates affecting the excess hazard and check whether the non-stratified version remains reliable under the null hypothesis. We try scenarios with balanced and imbalanced covariates, different number of strata and consider covariates that are or are not included in the population tables. Since sex is the only categorical variable in population tables, only one scenario with both *X*∈*D* and *S*∈*D* is considered here (variable *S* is age; it is categorized into three groups for the stratified version of the test). Further simulations exploring the number of strata used for stratification when both *X*∈*D* and *S*∈*D* can be found in Table 6. Since no important differences can be observed between the scenarios, we further study the power by picking only two of the scenarios in Table 4 with different number of strata and vary the size of the effect of *S*, these results are presented in Table 5.

**Table 4:** Comparison of the non-stratified and stratified log-rank type test for different covariate types: power

	62% events due to ex. haz.		93% events due to ex. haz.	
	*LRt*	*LRt-str*	*LRt*	*LRt-str*
*X*∈*D*, bal (sex); *S*∉*D*, bal, bin	0.498	0.501	0.879	0.882
*X*∈*D*, bal (sex); *S*∉*D*, imbal, bin	0.508	0.510	0.872	0.874
*X*∈*D*, bal (sex); *S*∉*D*, bal, 10 str	0.501	0.498	0.875	0.878
*X*∈*D*, bal (sex); *S*∉*D*, bal, bin, NPH	0.514	0.520	0.887	0.893
*X*∉*D*, bal, bin; *S*∈*D*, bal, bin (sex)	0.483	0.489	0.872	0.875
*X*∈*D*, bal (sex); *S*∈*D*, (age; 3 str)	0.512	0.513	0.871	0.873

Methods included: non-stratified (*LRt*) and stratified log-rank type test (*LRt-str*). ((im)bal = (im)balanced variable, i.e., the groups occur with (un)equal probabilities; bin= binary variable; 10 str = 10 strata; NPH = nonproportional effect). *X* is the categorical covariate of interest, *S* the stratification covariate, *D* denotes the demographic variables

**Table 5:** Comparison of the non-stratified and stratified log-rank type test for different effect sizes: power

	62% events due to ex. haz.		93% events due to ex. haz.	
	*LRt*	*LRt-str*	*LRt*	*LRt-str*
*X*∈*D*, bal (sex); *S*∉*D*, bal, bin, ef 0	0.505	0.505	0.870	0.870
*X*∈*D*, bal (sex); *S*∉*D*, bal, bin, ef 2x	0.502	0.519	0.863	0.880
*X*∈*D*, bal (sex); *S*∉*D*, bal, bin, ef 5x	0.465	0.559	0.778	0.894
*X*∈*D*, bal (sex); *S*∉*D*, bal, 10str, ef 0	0.495	0.496	0.879	0.875
*X*∈*D*, bal (sex); *S*∉*D*, bal, 10str, ef 2x	0.486	0.499	0.862	0.878
*X*∈*D*, bal (sex); *S*∉*D*, bal, 10str, ef 5x	0.472	0.559	0.772	0.895

Methods included: non-stratified (*LRt*) and stratified log-rank type test (*LRt-str*). (bal = balanced variable, i.e., the groups occur with equal probabilities; bin= binary variable; 10 str = 10 strata; ef= variable’s effect). *X* is the categorical covariate of interest, *S* the stratification covariate, *D* denotes the demographic variables

**Results** The size of both tests is very close to 0.05 with all the simulations performed (results are included in the Additional file 1), regardless of the number of strata and the size of the effect of *S*. This gives us confidence that the non-stratified version can be used reliably even if the hazards are non-homogeneous.

#### Non-stratified and stratified test - comparison of the power

**Comments on the simulation scenarios:** We repeat the same scenarios as in the previous subsection, but now with a non-zero effect of *X* (equal in all simulations). In Table 4 the effect sizes of *X* and *S* are comparable in size, in Table 5 the effect of *S* is varied.

**Results** The power of the stratified test tends to be larger than that of the non-stratified test, but the difference is not really striking (Table 4), the differences become important only when the effect of *S* is large compared to the effect of *X* (Table 5, other distributions of the covariates might bring larger differences). On the other hand, when there is no effect of *S*, no power seems to be lost by still performing the stratified test.

#### Stratified test - the effect of the number of strata

**Comments on the simulation scenarios:** As the last point, we further check the performance of the stratified test in case of many strata. To mimic a real life situation, we compare groups with respect to sex and stratify by age, which we can always expect to be an important factor. We simulate age as a continuous variable with a linear effect on log excess hazard, but then categorize it to allow for stratification. The effect of age is substantially larger than the effect of sex (5 times higher), so that some differences in power can be observed.

**Results** Table 6 once again confirms that the size of the non-stratified version of the test is appropriate and that the stratified test has more power. However, age is a continuous variable and thus the question is, how many strata to make. We can see that the power is best with 6 strata, but not much worse with only 3 strata, which is rather few considering that a strong effect of age was simulated. On the other hand, splitting to 30 strata which leaves some strata with only few events (practically all simulation runs include strata with less than 5 events), still provides an improved power compared to the non-stratified version. However, if the strata are far too many (last row of the Table 6), the power is importantly decreased. The reason is that many strata are without events or there is only one group within stratum and hence the information on excess hazard carried by the patients in these strata is not included in the estimation (on average a third of the patients belong to such strata).

**Table 6:** Comparison of the non-stratified and stratified log-rank type test for different number of strata

Length of age interval for stratification (no. of strata)	Size	Power
non-stratified	0.042	0.500
10 years (3 strata)	0.049	0.538
5 years (6 strata)	0.049	0.546
1 year (30 strata)	0.051	0.534
6 months (60 strata)	0.050	0.516
1 month (360 strata)	0.050	0.368

*X*∈*D* (sex is the categorical covariate of interest), *S*∈*D* (age (grouped) is the stratification covariate)

### Example

**Fig. 2:**
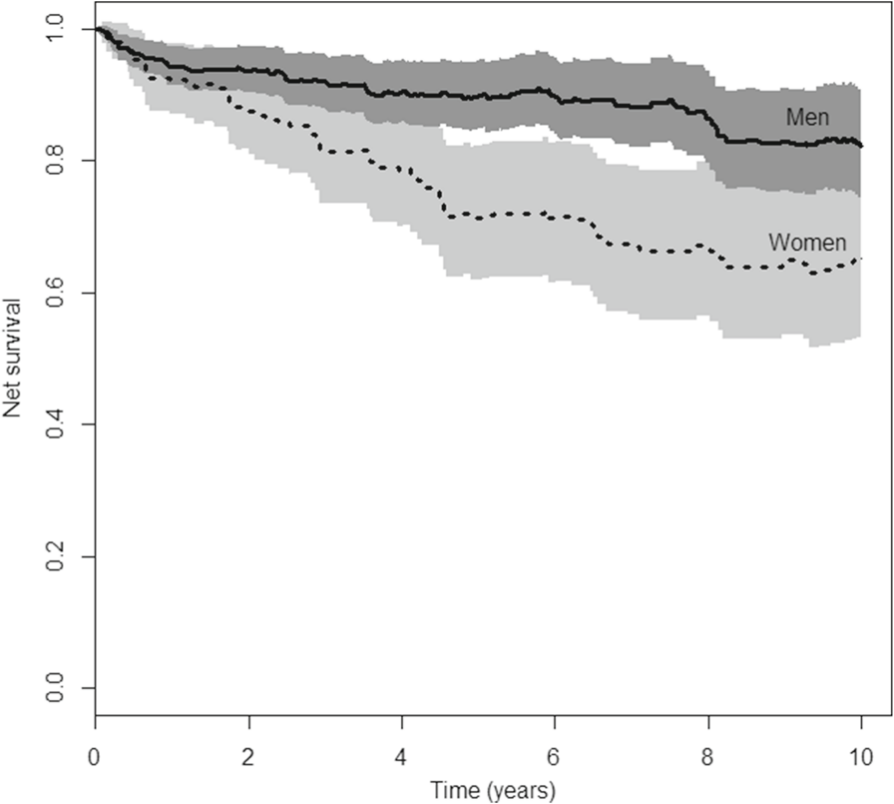
Comparison of net survival (PP estimator) for men and women

We return to our example on myocardial infarction. Out of 494 patients, 204 died (0.41). To get some idea of the power we can expect with our sample, we consider the proportion expected to die due to excess hazard (0.22; PP estimator) and the proportion expected to die in the population (0.31; population tables). The effect of sex in our sample is in the opposite direction as in the population, which, judging from the simulations, is also a positive indicator for the power. Figure 2 presents the net survival curves estimated by the PP estimator, we observe a marked difference between men and women that is confirmed by the log-rank type test with respect to sex (*p*=0.02).

To confirm our simulation results, we check also the semi-parametric and the fully parametric model (with one parameter for the baseline hazard in the first year of follow-up and another afterwards). Both yield equal interpretation, with *p* values 0.001 and 0.006, respectively.

Of course, both men and women differ in age (age span 45-75) and a univariate model including age shows that age is an important covariate in terms of excess hazard. If interested in the effect of sex conditional on age being the same, we can consider the stratified model. However, the stratification with respect to age cannot be very fine, a yearly stratification would imply that 10% of the patients (mostly the young ones) are omitted from the calculations.

Following our simulation results, we therefore limit ourselves to 2-year or 5-year strata, which results in *p* values equal to 0.18 and 0.09, respectively.

The different result than in the non-stratified test is expected as age and sex are not independent (women are on average 4.8 years older) and the usage of the stratified test thus implies a different interpretation. If we include age into a multivariate model and thus assume linearity of the effect, we get a significant effect of sex in the case of the semi-parametric model (*p*=0.03) and a borderline significant effect in the case of the fully parametric model (*p*=0.058). Since the linearity of age is hard to judge on our data (especially with the younger patients, where there are only a few individuals), the two models seem rather unreliable.

We can therefore conclude that the net survival curves differ significantly by sex, the non-stratified log-rank type test as a very reliable option can be used to show this. Whether or not this difference can be fully explained by the different age at infarction or persists within patients of same age, remains a question that is hard to respond to reliably with our sample, since the age-distribution is too wide for such a small sample and thus very little can be said about the youngest patients.

## Discussion

The inclusion of the log-rank type test into the arsenal of methods in relative survival may seem rather redundant at first, since the same hypothesis may be checked by regression modelling which can be directly generalized also to more complex problems. We have shown that the properties and the interpretation of the log-rank type test are in fact equal to those of the additive model. However, the test statistics are not identical and regression models require additional smoothing (semi-parametric models) or additional assumptions (fully parametric models). Therefore, the log-rank type test proves to be the simpler alternative that requires less assumptions (or tuning parameters), has a clearer form that lands itself to theoretical comparisons with other methods and at the same time gives very reliable results. The performance of the log-rank type test has been checked under many scenarios and no departures from the desired values were identified in terms of size. We have seen that the total sample size and the number of events do not provide direct information on the power of the log-rank type test. The key information is given by net mortality - the number of people that would die in the hypothetical world. On the other hand, the population mortality acts as the nuisance factor and lowers the amount of information in the real world.

When introduced in [1], two versions of the test statistic for the log-rank type test were proposed. While it is true that the assumption of hazard homogeneity is required for the log-rank type test in theory, we have found no scenario where the non-stratified version would not remain reliable under the null hypothesis even if this assumption is not met. This is consistent with the theory in the hypothetical world, where we know that omitting a covariate in the Cox model does not have an important effect on the size of the test [15] and hence the same is true for the log-rank test. Note however that, while all the crucial parameters that could affect the change of properties between the hypothetical and real world were considered in the simulations, one cannot use these simulations as an indication that the size of the semi-parametric additive model is reliable, since the additional assumptions of this test (smoothing) were not addressed in our simulations. Under the alternative hypothesis, when the net survival curves truly differ, the power of the non-stratified test is lower and may be importantly lower if the effect of the ignored variable that causes inhomogeneity is high. On the other hand, some loss of power can also be observed when the data are overly stratified and overly small groups are formed, but we have shown that as long as a reasonable amount of stratification is performed, no important loss of power can be expected. While this result may lead to the conclusion, that the stratified model is a sensible choice, this is in fact true only for independent covariates, a fact that cannot be checked in practice. When covariates are not independent, the interpretation of the stratified test is conditional on the stratification covariate and thus importantly different from the interpretation of the non-stratified: the non-stratifed version tests whether a covariate *X* is related to survival, the stratified version tests whether a covariate *X* is related to survival in patients with equal values of variable *S*. Therefore, the stratified version of the test should not be understood as the alternative with the better power, but rather as a test that addresses a different research question.

## Conclusions

The log-rank type test presents a stable and reliable tool for comparing net survival between groups, which requires less assumptions than its alternative, the additive regression model. No scenarios presenting departures from the nominal size could be identified. The power of the test depends on the total sample size and the number of events, with the number of events of interest being the crucial factor determining power and the number of other-cause deaths serving as a nuisance factor. The properties of the test remain favorable also in the case of non-homogeneous hazards, its stratified version can be used if comparisons conditional on a second covariate are of interest. The interpretation of both the log-rank type test and its stratified version equals to that of an analogous semi-parametric additive regression model despite the fact that no direct theoretical link can be established between the test statistics.

## Additional file

Additional file 1 Supplementary tables. Additional simulation results. The file includes two tables containing the estimated size of both the non-stratified and stratified version of the log-rank type test in different scenarios (with 62\% or 93\% of events due to excess hazard). Size is close to the nominal value of 0.05 in all cases. Additionally, results for the simulations with proportion of excess hazard death equal to 41% are included. They exhibit similar behaviour, both versions of the log-rank type test seem reliable. (PDF 58 kb)

## Abbreviations

AMI: Acute myocardial infarction
bin: Binary variable
ef: Variable’s effect
fAM: Fully-parametric additive model
grps: Groups
(im)bal: (im)balanced variable
LRh: Log-rank test on the hypothetical world data
LRt: Log-rank type test on the real world data
NPH: Nonproportional hazard
PP: Pohar Perme estimator
sAM: Semi-parametric additive model
str: Strata, stratified
