# Supplementary material for: On comparison of net survival curves
Source: BMC Med Res Methodol. 2017 May 2;17:79. doi: 10.1186/s12874-017-0351-3 (PMC5414380; doi:10.1186/s12874-017-0351-3)
Supplement: Additional file 1 — Supplementary tables. Additional simulation results. The file includes two tables containing the estimated size of both the non-stratified and stratified version of the log-rank type test in different scenarios (with 62\% or 93\% of events due to excess hazard). Size is close to the nominal value of 0.05 in all cases. Additionally, results for the simulations with proportion of excess hazard death equal to 41% are included. They exhibit similar behaviour, both versions of the log-rank type test seem reliable. (PDF 58 kb) [file 12874_2017_351_MOESM1_ESM.pdf]

Additional file 1

Supplementary tables

Table S1: Comparison of the non-stratified and stratified log-rank type test for different covariate types: size. Methods included: non-stratified (*LRt*) and stratified log-rank type test (*LRt-str*). (*LRh*). ((im)bal = (im)balanced variable, i.e., the groups occur with (un)equal probabilities; bin= binary variable; 10 str = 10 strata; NPH = nonproportional effect). X is the categorical covariate of interest, S the stratification covariate, D denotes the demographic variables.

	62% events due to ex. haz.		93% events due to ex. haz.	
	LRt	*LRt-str*	*LRt*	*LRt-str*
$X \in D$, bal (sex); $S \notin D$, bin, bal	0.051	0.051	0.053	0.052
$X \in D$, bal (sex); $S \notin D$, bin, imbal	0.047	0.048	0.048	0.048
$X \in D$, bal (sex); $S \notin D$, bal, 10 str	0.051	0.050	0.050	0.053
$X \in D$, bal (sex); $S \notin D$, bin, bal, NPH	0.051	0.050	0.050	0.050
$X \notin D$ bin, bal; $S \in D$, bal (sex)	0.045	0.043	0.048	0.047
$X \in D$, bal (sex); $S \in D$, (age; 3 str)	0.050	0.051	0.051	0.052

Table S2: Comparison of the non-stratified and stratified log-rank type test for different effect sizes: size. Methods included: non-stratified (*LRt*) and stratified log-rank type test (*LRt-str*). (bal = balanced variable, i.e., the groups occur with equal probabilities; bin= binary variable; 10 str = 10 strata; ef= variable's effect). X is the categorical covariate of interest, S the stratification covariate, D denotes the demographic variables.

	62% events due to ex. haz.		93% events due to ex. haz.	
	LRt	*LRt-str*	*LRt*	*LRt-str*
$X \in D$, bal (sex); $S \notin D$, bal, bin, ef 0	0.051	0.049	0.046	0.045
$X \in D$, bal (sex); $S \notin D$, bal, bin, ef 2x	0.050	0.049	0.046	0.047
$X \in D$, bal (sex); $S \notin D$, bal, bin, ef 5x	0.048	0.046	0.050	0.053
$X \in D$, bal (sex); $S \notin D$, bal, 10str, ef 0	0.050	0.049	0.052	0.052
$X \in D$, bal (sex); $S \notin D$, bal, 10str, ef 2x	0.048	0.047	0.046	0.050
$X \in D$, bal (sex); $S \notin D$, bal, 10str, ef 5x	0.049	0.050	0.047	0.047

Table S3: Comparison of the log-rank type test and the additive model: size. Methods included: log-rank type (*LRt*), semi-parametric additive model (*sAM*), fully parametric additive model (*fAM*), log-rank test in hypothetical world (*LRh*). ((im)bal = (im)balanced variable, i.e., the groups occur with (un)equal probabilities; bin= binary variable; 4 grps = a variable with four groups). X is the categorical covariate of interest, D denotes the demographic variables.

	41 % events due to ex. haz.			
	LRt	*sAM*	*fAM*	*LRh*
$X \in D$, bal, bin (sex)	0.046	0.071	0.047	0.051
$X \notin D$, bal, bin	0.051	0.066	0.045	0.048
$X \notin D$, imbal, bin	0.057	0.054	0.049	0.047
$X \notin D$, bal, 4 grps	0.050	0.067	0.035	0.055

Table S4: Comparison of the log-rank type test and the additive model: power. Methods included: log-rank type (*LRt*), semi-parametric additive model (*sAM*), fully parametric additive model (*fAM*), log-rank test in hypothetical world (*LRh*). ((im)bal = (im)balanced variable, i.e., the groups occur with (un)equal probabilities; bin= binary variable; 4 grps = a variable with four groups; ef = variable's effect; NPH = nonproportional effect). X is the categorical covariate of interest, D denotes the demographic variables.

	41 % events due to ex. haz.			
	LRt	*sAM*	*fAM*	*LRh*
$X \in D$, bal, bin (sex)	0.224	0.314	0.267	0.649
$X \notin D$, bal, bin	0.216	0.355	0.315	0.648
$X \notin D$, imbal, bin, ef > 0	0.199	0.036	0.008	0.522
$X \notin D$, imbal, bin, ef < 0	0.230	0.506	0.474	0.760
$X \notin D$, bal, 4 grps	0.152	0.24	0.167	0.473
$X \notin D$, bal, bin, NPH, ef ≈ 0	0.052	0.090	0.061	0.052
$X \notin D$, bal, bin, NPH	0. 210	0.269	0.204	0.647

Table S5: Comparison of the non-stratified and stratified log-rank type test for different covariate types: size. Methods included: non-stratified (*LRt*) and stratified log-rank type test (*LRt-str*). (*LRh*). ((im)bal = (im)balanced variable, i.e., the groups occur with (un)equal probabilities; bin= binary variable; 10 str = 10 strata; NPH = nonproportional effect). X is the categorical covariate of interest, S the stratification covariate, D denotes the demographic variables.

	41 % events due to ex. haz.	
	LRt	*LRt-str*
$X \in D$, bal (sex); $S \notin D$, bal, bin	0.051	0.052
$X \in D$, bal (sex); $S \notin D$, imbal, bin	0.047	0.049
$X \in D$, bal (sex); $S \notin D$, bal, 10 str	0.047	0.047
$X \in D$, bal (sex); $S \notin D$, bal, bin, NPH	0.050	0.049
$X \notin D$, bal, bin; $S \in D$, bal, bin (sex)	0.050	0.049
$X \in D$, bal (sex); $S \in D$, (age; 3 str)	0.049	0.049

Table S6: Comparison of the non-stratified and stratified log-rank type test for different covariate types: power. Methods included: non-stratified (*LRt*) and stratified log-rank type test (*LRt-str*). (*LRh*). ((im)bal = (im)balanced variable, i.e., the groups occur with (un)equal probabilities; bin= binary variable; 10 str = 10 strata; NPH = nonproportional effect). X is the categorical covariate of interest, S the stratification covariate, D denotes the demographic variables.

	41 % events due to ex. haz.	
	LRt	*LRt-str*
$X \in D$, bal (sex); $S \notin D$, bal, bin	0.215	0.217
$X \in D$, bal (sex); $S \notin D$, imbal, bin	0.221	0.222
$X \in D$, bal (sex); $S \notin D$, bal, 10 str	0.228	0.230
$X \in D$, bal (sex); $S \notin D$, bal, bin, NPH	0.224	0.227
$X \notin D$, bal, bin; $S \in D$, bal, bin (sex)	0.221	0.222
$X \in D$, bal (sex); $S \in D$, (age; 3 str)	0.218	0.221

Table S7: Comparison of the non-stratified and stratified log-rank type test for different effect sizes: size. Methods included: non-stratified (*LRt*) and stratified log-rank type test (*LRt-str*). (bal = balanced variable, i.e., the groups occur with equal probabilities; bin= binary variable; 10 str = 10 strata; ef= variable's effect). X is the categorical covariate of interest, S the stratification covariate, D denotes the demographic variables.

	41 % events due to ex. haz.	
	LRt	*LRt-str*
$X \in D$, bal (sex); $S \notin D$, bal, bin, ef 0	0.044	0.044
$X \in D$, bal (sex); $S \notin D$, bal, bin, ef 2x	0.049	0.048
$X \in D$, bal (sex); $S \notin D$, bal, bin, ef 5x	0.052	0.051
$X \in D$, bal (sex); $S \notin D$, bal, 10str, ef 0	0.052	0.050
$X \in D$, bal (sex); $S \notin D$, bal, 10str, ef 2x	0.047	0.046
$X \in D$, bal (sex); $S \notin D$, bal, 10str, ef 5x	0.051	0.051

Table S8: Comparison of the non-stratified and stratified log-rank type test for different effect sizes: power. Methods included: non-stratified (*LRt*) and stratified log-rank type test (*LRt-str*). (bal = balanced variable, i.e., the groups occur with equal probabilities; bin= binary variable; 10 str = 10 strata; ef= variable's effect). X is the categorical covariate of interest, S the stratification covariate, D denotes the demographic variables.

	41 % events due to ex. haz.	
	LRt	*LRt-str*
$X \in D$, bal (sex); $S \notin D$, bal, bin, ef 0	0.224	0.224
$X \in D$, bal (sex); $S \notin D$, bal, bin, ef 2x	0.235	0.237
$X \in D$, bal (sex); $S \notin D$, bal, bin, ef 5x	0.235	0.264
$X \in D$, bal (sex); $S \notin D$, bal, 10str, ef 0	0.223	0.220
$X \in D$, bal (sex); $S \notin D$, bal, 10str, ef 2x	0.223	0.229
$X \in D$, bal (sex); $S \notin D$, bal, 10str, ef 5x	0.245	0.271

Table S9: Comparison of the non-stratified and stratified log-rank type test for different number of strata. $X \in D$ (sex is the categorical covariate of interest), $S \in D$ (age (grouped) is the stratification covariate). 41 % of events are due to excess hazard.

length of age interval for stratification (no. of strata)	size	power
non-stratified	0 .047	0.226
10 years (3 strata)	0.051	0.248
5 years (6 strata)	0.051	0.246
1 year (30 strata)	0.054	0.240
6 months (60 strata)	0.051	0.233
1 month (360 strata)	0.049	0.174
